# A new source of bacterial myrosinase isolated from endophytic *Bacillus* sp. NGB-B10, and its relevance in biological control activity

**DOI:** 10.1007/s11274-022-03385-3

**Published:** 2022-09-03

**Authors:** Sameh H. Youseif, Hanan M. K. Abdel-Fatah, Mary S. Khalil

**Affiliations:** 1 Department of Microbial Genetic Resources, National Gene Bank, Agricultural Research Center (ARC), Giza, 12619 Egypt; 2 Faculty of Biotechnology, October University for Modern Sciences and Arts (MSA), 6th October, Giza, 12451 Egypt; 3 Department of Botany and Microbiology, Faculty of Science, Cairo University, Giza, 12613 Egypt

**Keywords:** *Bacillus* sp., Microbial myrosinase, Glucosinolates, Glycoside hydrolase, Response surface methodology, Antifungal activities

## Abstract

Plant metabolism interacts strongly with the plant microbiome. Glucosinolates, secondary metabolites synthesized by *Brassica* plants, are hydrolyzed by myrosinase into bioactive compounds of great importance in human health and plant protection. Compared with myrosinase from plant sources, myrosinase enzymes of microbial origin have not been extensively investigated. Therefore, seven endophytic strains corresponding to *Bacillus* sp. were isolated from *Eruca vesicaria* ssp*. sativa* plants that could hydrolyse glucosinolates (sinigrin) in the culture medium and showed myrosinase activity (0.08–19.92 U mL^−1^). The *bglA* myrosinase-related gene encoding the 6-phospho-β-glucosidase (GH 1) from *Bacillus* sp. NGB-B10, the most active myrosinase-producing bacterium, was successfully identified. Response surface methodology (RSM) was applied to statistically optimize culture conditions for myrosinase production from *Bacillus* sp. strain NGB-B10. The Plackett–Burman design indicated that nitrogen concentration, incubation period, and agitation speed were the significant parameters in myrosinase production. The application of the Box–Behnken design of RSM resulted in a 10.03-fold increase in enzyme activity as compared to the non-optimized culture conditions. The myrosinase was partially purified by 40% fractionation followed by SDS-PAGE analysis which yielded two subunits that had a molecular weight of 38.6 and 35.0 KDa. The purified enzyme was stable under a broad range of pH (5.5–10) and temperatures (10–65 °C). The hydrolysis products released by bacterial myrosinase from some glucosinolate extracts had higher and/or equivalent in vitro antagonistic activity against several phytopathogenic fungi compared to the nystatin (a broad-spectrum antifungal agent). This study provides original information about a new source of bacterial myrosinase and affords an optimized method to enhance myrosinase production.

**Supplementary Information:**

The online version contains supplementary material available at 10.1007/s11274-022-03385-3.

## Introduction

**Fig. 1:**
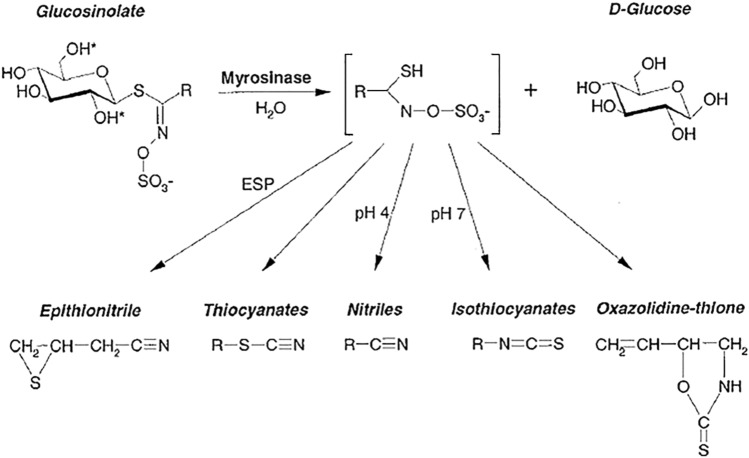
Scheme of enzymatic hydrolysis of GLS and their derivatives (Rask et al. 2000)

Glucosinolates (GLs) are an important class of sulfur-containing secondary metabolites that are present exclusively in plants of the Brassicales, mainly in the family *Brassicaceae* (*Cruciferae*) such as cabbage, broccoli, mustard, arugula, and cauliflower (Bhat and Vyas 2019). Generally, GLs are stored in the plant vacuole or specific S-cells, and upon tissue disruption, they are hydrolyzed by the myrosinase enzyme (β-thioglucoside glucohydrolase EC 3.2.3.1), which is stored in so-called myrosin cells separately from the GLs, into D-glucose and unstable intermediates (Martinez-Ballesta and Carvajal 2015; Wittstock et al. 2016). These intermediates spontaneously rearrange into isothiocyanates, thiocyanates, nitriles, epithionitriles, or oxazolidine-2-thiones (Fig. 1) depending on the substrate, reaction conditions (such as pH values, temperature), and presence of certain proteins (Hanschen et al. 2017; Sikorska-Zimny and Beneduce 2021).

The GLs-myrosinase system not only plays a significant role in the plant’s defense against biotic stressors but also contributes to beneficial effects for human health and plant protection. GLs derivatives particularly isothiocyanates (ITCs) are effective candidates against several herbivores, pests, and pathogens as these compounds have antifungal, antimicrobial as well as insecticidal activities (Wittstock et al. 2016; Wassermann et al. 2017; Bhat and Vyas 2019). Due to their high antimicrobial properties and natural plant origin, ITCs also have been previously studied in the food industry and preservation (Dufour et al. 2015; Yaqoob et al. 2020). For humans, the biological activities of GLs and their breakdown products have been well documented in the literature for their disease-preventive and therapeutic effects (Romeo et al. 2018). Several studies have described the protective effects of ITCs against various types of cancer (Rudolf et al. 2014; Lawson et al. 2015) in addition to their positive effect on cardiovascular and neurological diseases (Palliyaguru et al. 2018).

Myrosinase has so far been well-characterized in several plant genera (Bhat and Vyas 2019). However, myrosinase has been also identified in insects (Jones et al. 2001; Pontoppidan et al. 2001), fungi (Rakariyatham et al. 2005; Szucs et al. 2018; Abdel-Fatah et al. 2021), and human gut microbiota (Liou et al. 2020; Sikorska-Zimny and Beneduce 2021). Many reports have described the isolation and characterization of myrosinase-producing strains belonging to diverse bacterial genera such as *Bacillus* (El-Shora et al. 2016; Luang-In et al. 2018), *Companilactobacillus* (Watanabe et al. 2021) *Citrobacter* (Albaser et al. 2016; Cebeci et al. 2022), *Enterobacter* (Tani et al. 1974; Wassermann et al. 2017; Luang-In et al. 2018), *Enterococcus* (Luang-In et al. 2016, 2018)*, Lactobacillus* (Palop et al. 1995; Luang-In et al. 2014, 2016), *Lactococcus* (Mullaney et al. 2013; Luang-In et al. 2018), and *Leclercia* (Tie et al. 2021) which are capable of in-vitro metabolism of GLs. Previous studies reported that the enzyme 6-phospho-β-glucosidase, encoded by the genes *bglA*, *ascB*, and *chbF*, is associated with myrosinase-like activity in bacteria such as *Escherichia coli* strain O157:H7 (Cordeiro et al. 2015) and *Enterobacter cloacae* strain KS50 (Wassermann et al. 2017)*.*

For the efficient production of bacterial enzymes, optimizing media components and culture conditions is very important which affects the economics and feasibility of the process (Ojha et al. 2020). Recently, response surface methodology (RSM) has been widely used as a mathematical modeling and a statistical tool for optimizing conditions in multivariable systems, while saving time and labor by minimizing the number of required experiments (Mefteh et al. 2019; Abdel-Rahman et al. 2020; Zhao et al. 2020). Box–Behnken Design (BBD) is one of the main experimental popular designs used in the RSM for optimizing the production of bacterial enzymes (Tabssum et al. 2018; Mefteh et al. 2019).

The plant-endophytic microbiome is considered a major source of secondary metabolites and bioactive natural products. Endophytic bacteria are ubiquitous and it is now well established that the individual plant hosts one or more endophytes (Gouda et al. 2016). Recently, several studies reported that endophytes and their hosts contribute partially to the metabolism of each other (Naik et al. 2019; Hagaggi and Mohamed 2020) and therefore some metabolites are co-products of the endophytes and the plants (Ludwig-Müller 2015). For example, a recent matching of bioactive secondary metabolites (total phenolic content, flavonoids, saponins, and tannins) production was identified between *Calotropis procera* and its associated bacterial endophytes (Hagaggi and Mohamed 2020).

Therefore, we hypothesized that due to their high GLs content, *E. vesicaria* ssp*. sativa* (Mill.) Thell*.* (arugula) plants belonging to the family *Brassicaceae* may harbor a specific microbiome containing potential myrosinase-producing bacteria. We also raised the following questions: Do endophytes have a potential myrosinase activity better than non-endophytic “rhizospheric” bacteria? What are the optimum culture conditions for myrosinase production? To which extent do GLs-myrosinase hydrolysis products have antagonistic activities against plant pathogenic fungi? To answer the above questions, rhizospheric and endophytic bacteria associated with *E. vesicaria* ssp*. sativa* plants were isolated and screened for GLs-degradation activities. GLs-degrading bacteria were identified using 16S rRNA sequence analysis and the sequence of the myrosinase-related gene (*bglA*) was determined. The culture conditions for myrosinase production by the highest producing bacterium (*Bacillus* sp. NGB-B10) were optimized using combined statistical approaches including Plackett–Burman experiment, and the BBD of RSM. The partially purified enzyme was also characterized to study enzyme biochemical characteristics and address its kinetic properties. Moreover, the antagonistic effect of several sources of GLs-hydrolysis products against phytopathogenic fungi was investigated.

## Materials and methods

### Bacterial isolation

Ten plant samples of *E. vesicaria* ssp*. sativa* (Mill.) Thell. (arugula) were collected from the Agricultural Experimental and Research Station (Latitude 30.026455, Longitude 31.192168), Faculty of Agriculture, Cairo University. Rhizospheric bacteria was isolated as described in (Youseif 2018). Soil adhering with plant roots was collected and mixed. Then, 10 gm was taken and a serial dilution was made and plated onto nutrient agar (NA) plates (0.5% peptone, 0.15% yeast extract, 0.15% beef extract, 0.5% sodium chloride, and 1.5% agar). Endophytic bacteria were obtained from surface-sterilized plant tissues as described in (Hallmann et al. 2006). Root and leave samples were separated and surface-sterilized by washing for 2 min in 70% ethanol, immersed in 1.5% sodium hypochlorite for 3 min, and finally washed 3 times using sterile distilled water under aseptic conditions. Surface sterilization efficiency was tested by imprinting the surface-sterilized plant tissue onto NA plates and spreading aliquots of the last rinse wash on the same type of medium as described in (Youseif et al. 2021a). Individual leaves and roots were cut into small pieces and aseptically ground in sterile distilled water, streaked on the surface of NA plates supplemented with 25 µg mL^−1^ nystatin, and finally were incubated at 30 °C for 5–7 days. The bacterial colonies were initially screened and grouped by colony color and morphological characteristics.

### Screening for myrosinase-active bacteria (GLs-degrading organisms)

The activity of bacterial isolates to produce myrosinase and decompose GLs was assessed as described in (Tani et al. 1974) using a mustard extract broth medium (0.01% sinigrin, 6% mustard extract, 0.1% KH_2_PO_4_, 0.1% NH_4_Cl, 0.1% NaCI, and 0.1% MgSO_4_·7H_2_O), all listed chemicals were purchased from Sigma-Aldrich, Merck KGaA, Germany. Bacterial isolates were inoculated in a mustard extract medium and incubated for 48 h. Then, cultures were centrifuged at 6000 rpm for 10 min at 4 °C and the supernatants were screened for GLs breakdown based on the barium sulfate test as described by Sakorn et al. (2002). Myrosinase activity was determined according to Palmieri et al. (1982). One unit of myrosinase activity was defined as the amount of enzyme capable of hydrolyzing 1 μmol of sinigrin per minute per mL under the experimental conditions.

### Molecular identification and phylogenetic analysis

Total genomic DNA of bacterial cells showing myrosinase activity was isolated and purified using GeneJet™ Genomic DNA purification Kit (Thermo Scientific®, Waltham, USA) according to the manufacturer’s protocol. The 16S rRNA gene was amplified using 27f and 1492r universal oligonucleotides (Weisburg et al. 1991). PCR was performed using the standard reaction mixture as described in (Youseif 2018). PCR reaction conditions were: initial denaturation at 95 °C for 3 min, 35 cycles of denaturation at 94 °C for 1 min, annealing at 58 °C for 45 s, and extension at 72 °C for 2 min. PCR fragments were purified using QIAquick PCR Purification Kit (Qiagen, Hilden, Germany) and sent to a sequencing service (Macrogen, Seoul, South Korea). Sequence reads were edited and assembled using DNA Baser assembly software (www.DnaBaser.com). Isolates were identified by comparing their 16S rRNA sequences with reference sequences available at Ezbiocloud (https://www.ezbiocloud.net/) and GenBank (www.ncbi.nlm.nih.gov) databases as previously described in (Youseif et al. 2021b). The sequences were aligned using ClustalW matrix-based algorithm and were subjected to phylogenetic analyses using MEGA X software version 10 (Kumar et al. 2018). 16S rRNA phylogenetic tree was generated using the Maximum likelihood (ML) algorithm and p-distance model.

### Amplification of myrosinase-related sequence

The 6-phospho-beta-glucosidase (*bglA*) myrosinase-related gene was amplified from the highest myrosinase-producing bacterium (NGB-B10). To amplify myrosinase-related sequence, forward (5′- TTTATGGGGAGGCGCTCTAG-3′) and reverse (5′-GCGTTCAAACACACTTCAGC-3′) primers were designed based on sequence similarities of *bglA* gene from *E. coli* O157:H7 strain TW14359 (accession: CP001368.1) and *Enterobacter ludwigii* strain EcWSU1 (accession: CP002886.1) which had been identified as myrosinase-producing organisms (Cordeiro et al. 2015). The myrosinase-related sequences were retrieved from the GenBank database (www.ncbi.nlm.nih.gov). Primers were designed using BioEdit sequence alignment editor version 7.2 (https://bioedit.software.informer.com/) and Primer3 version 4.1 software (https://primer3.ut.ee/). PCR conditions were set to 95 °C, 3 min; 30 cycles of 95 °C, 30 s; 63 °C, 30 s, 72 °C, 30 s; and a final extension at 72 °C, 10 min. PCR product was then purified using QIAquick Gel Extraction Kit (Qiagen, Hilden, Germany) and sent to a sequencing service (Macrogen, Seoul, South Korea).

### Media optimization by one factor at a time (OFAT) method

Optimal media components for myrosinase production by *Bacillus* sp. strain NGB-B10 were screened via the OFAT method. The experiments were conducted in 500 mL Erlenmeyer flasks containing 200 mL using a mustard extract broth medium. After autoclaving, the flasks were cooled and inoculated with 1 mL freshly-grown culture containing 10^6^ CFU mL^−1^. All experiments were conducted in three sets and data were presented as mean values with ± SD.

For carbon sources, the leaf extract of *Eruca vesicaria* ssp*. sativa* (Mill.) Thell. (arugula), *Brassica oleracea* L*.* (red cabbage and white cabbage), *Raphanus sativus* L*.* (red radish and white radish); root extract of red radish and white radish; and seed extract of *Sinapis alba* L. (white mustard) and *Brassica nigra* (L.) W.D.J. Koch (black mustard seeds) were selected to evaluate the effect of carbon sources as inducers of myrosinase activity. The taxonomy and nomenclature were done according to The Integrated Taxonomic Information System (ITIS, https://www.itis.gov/). Plant samples were purchased from markets, stored at − 80 °C for 24 h, and then freeze-dried. The natural GLs extracts were prepared from cruciferous plants according to the hot methanol extraction method (ISO 9167-1) as previously described in (Ishida et al. 2011). A 20 g of freeze-dried plant sample was grinding and cooked in hot water for 25 min. Then, the cooked samples were homogenized with methanol in a 3:2 solvent to fresh weight ratio and subsequently boiled at 80 °C under reflux for 30 min. The extract was filtered then methanol was evaporated in a rotary evaporator. The residual extract of the GLs extract was dissolved in 50 mL sterile 0.05 M phosphate buffer (pH 7). Each carbon source was investigated at a concentration of 2% and 10% (w/v) in the production medium.

For nitrogen sources, a concentration of 200 mmol L^−1^ of sodium nitrate, potassium nitrate, ammonium nitrate, ammonium chloride, and ammonium sulfate was selected to study the effect of different inorganic nitrogen sources on myrosinase activity.

### Plackett–Burman statistical design for effective constituents

Seven independent variables including essential media components and culture conditions were selected for Plackett–Burman design (PBD) analysis as follows; agitation, C-source concentration, N-source concentration, temperature, pH, incubation period, and inoculum size. These factors were tested in 12 experimental runs and each factor was investigated at 2 levels; low level (−) and high level (+) as illustrated in Table S1. One millimeter freshly-grown culture of *Bacillus* sp. strain NGB-B10 containing 10^6^ CFU mL^−1^ was inoculated into the production media and incubated at 30 °C for 48 h. PBD is based on the first-order model:

$${\text{Y}} = \beta {\text{o}} + \sum \beta i{\text{X}}i.$$

where Y is the response (myrosinase production), β0 is the model intercept and βi is the linear coefficient, and Xi is the level of the independent variable. The statistical discovery JMP software version 14.1 (www.jmp.com) was used for the Plackett–Burman experimental design and subsequent regression analysis. The experiments were carried out in 3 replicates and the response was calculated as the average value of the results. Regression analysis was performed and significant variables were further optimized by using the RSM design.

### The Box Behnken experimental design

According to PBD results, the BBD of RSM was employed to optimize the most significant variables (nitrogen concentration, incubation period, and agitation speed) for enhancing myrosinase production. Each variable was analyzed at 3 levels, low (− 1), medium (0), and high (+ 1) levels (Table S2). The design of the matrix includes 15 trials, all the trials were performed in triplicates and the final response value was calculated as the mean of three replicates (± SD). Myrosinase production was fitted using a second-order polynomial equation for predicting the optimal point. The general form of the second-order polynomial equation is:

$$Y = \beta_{0} + \sum \beta_{i} X_{i} + \sum \beta_{ij} X_{i} X_{j} + \sum \beta_{ii} X_{i}^{2}$$

where Y is the predicted response, β0 is the intercept term, βi is the linear coefficient, βij is the quadratic coefficient, βii is the interaction coefficient, and XiXj represents the independent variables. The fitness of the polynomial model equation was evaluated by the coefficient of determination *R*^*2*^ and adjusted *R*^*2*^*.*

### Purification of the myrosinase enzyme

The crude extract of myrosinase was prepared using a modified M9 medium (10% leaf extract of red cabbage, 0.2% NaNO_3_, 0.3% KH_2_PO_4_, 0.05% MgSO_4_·7H_2_O, and 0.002% CaCl_2_·6H_2_O) as revealed by the statistical media optimization. One-millimeter fresh culture (OD_600_ of 1.0) of *Bacillus* sp. strain NGB-B10 was inoculated into a 2 L flask containing 1 L of autoclaved medium and incubated in a shaking incubator at 250 rpm for 12 h at 30 °C. Bacterial culture was centrifuged at 6,000 rpm at 4 °C for 15 min and myrosinase activity was detected in the supernatant as described in (Palmieri et al. 1982). The total protein amount was determined by measuring optical density at 280 nm (Layne 1957; Stoscheck 1990).

The myrosinase enzyme was purified and precipitated using ammonium sulfate fractionation. The crude extract was subjected to 20, 40, 60, 80, and 100% ammonium sulfate saturation by adding the salt slowly under continuous magnetic stirring in an ice water bath and was kept overnight at 4 °C. The number of grams of ammonium sulfate for each fraction was calculated using the online calculator (https://www.encorbio.com/protocols/AM-SO4.htm). After the precipitation of each fraction, the precipitated protein was separated by centrifugation at 15,000 rpm at 4 °C for 15 min (Kapilan 2016). The obtained pellet was dissolved in 15 mL of 0.1 M phosphate buffer pH 7.0 and dialyzed against the same buffer overnight at 4 °C using Spectrum™ dialysis membrane (Thermo Fisher Scientific, Waltham, USA). The dialysis process was repeated till ammonium sulfate was completely desalted. After dialysis, myrosinase activity and protein content in each fraction was determined as abovementioned.

The subunits of purified myrosinase enzyme in the 40% fraction with the highest activity were determined by using a sodium dodecyl sulfate–polyacrylamide gel electrophoresis SDS-PAGE as described in (Mohammed et al. 2008). SDS-PAGE analysis was performed using 12% acrylamide separating gel and 4% acrylamide stacking gel on a Mini-PROTEIN II Cell electrophoresis unit (Bio-Rad, Hercules, CA). Protein bands were visualized by staining with coomassie blue R-250. GangNam˗Stain™ prestained protein ladder was used as a standard (iNtRON Biotechnology, South Korea).

### Characterization of the partially purified myrosinase enzyme

The purified extract of 40% fraction with the highest enzymatic activity was used to determine the main characteristics of the enzyme. All the assays were performed in triplicate. One hundred microliters of 2 mM of sinigrin dissolved in 0.05 M phosphate buffer pH 7.0 were incubated with 20 µl of pure enzyme extract. The enzyme activity was determined by incubating the reaction mixture at different temperatures in the range from 10 to 90 °C. The optimum temperature for myrosinase activity was calculated as previously described in (Palmieri et al. 1982) by plotting enzyme activity against temperatures. The effect of pH on myrosinase activity was measured in buffers at different pH values ranging from 3.0 to 7.0 (using 0.05 mM sodium citrate buffer), 7.5 to 8.0 (using 0.05 mM sodium phosphate buffer), and 8.6 to 10.6 (using 0.05 mM glycine–NaOH buffer). The velocity of sinigrin catalysis by myrosinase was determined by using different concentrations of substrate (1, 2, 5, 10, 15, and 20 mM at 0.05 M in phosphate buffer pH 7.0). Enzyme kinetic constants (K_m_ and V_max_) were determined according to the double-reciprocal Lineweaver–Burk plot methods.

### Screening the antifungal activity of GLs-myrosinase hydrolysis products

The antifungal activity of the GLs-hydrolysis products was evaluated against 9 phytopathogenic fungal strains corresponding to the following genera: *Alternaria*, *Aspergillus*, *Curvularia*, *Fusarium*, *Penicillium*, *Sclerotium*, and *Stemphylium* by using the agar well diffusion method*.* The plant pathogenic taxa were provided from the Suez Canal University Fungarium (SCUF), Egypt (https://ccinfo.wdcm.org/collection/by_id/1180). GLs natural extracts from 7 *Brassica* plants were hydrolyzed by adding myrosinase purified from *Bacillus* sp. NGB-B10. Cruciferous plant samples were purchased from markets and GLs extracts were prepared using the hot methanol extraction method (ISO 9167–1) as abovementioned. A 10 U of the purified myrosinase was added to them and incubated at 37 °C for 60 min to release hydrolytic products. Five hundred microliters of freshly-grown fungal cultures (10^8^ spores mL^−1^) were spread on Czapek Dox agar plates (0.2% NaNO_3_, 0.05% KCl, 0.1% K_2_HPO_4_, 0.05% MgSO_4_, 0.001% FeSO_4_, 3% sucrose, and 1.5% agar). On each plate, wells were punched in the medium using a 6 mm diameter sterilized borer. Then, 0.5 mL of GLs-myrosinase complex was added to the wells, and subsequently, the plates were kept for 3˗4 days at 30 °C. A well containing 0.5 mL nystatin (25 µg mL^−1^) was used as positive control while 0.5 mL of each extract and 10 U of myrosinase were served as individual negative controls. The experiments were carried out in triplicates and antifungal activities were expressed as the mean of the inhibition zone diameters (mm).

### Statistical analyses

The data obtained were expressed as mean ± standard deviation (SD). The statistical significance of the model was estimated using a one-way analysis of variance (ANOVA) at a significance level of 5% (*p* > 0.05) using the statistical analysis software SPSS version 16.0 (www.ibm.com). The mathematical modeling was performed using the statistical discovery JMP software version 14.1 (www.jmp.com).

## Results

### Isolation of myrosinase-producing bacteria

A total of 36 bacterial isolates (15 rhizospheric and 21 endophytic) were isolated in this study. The endophytic bacteria were isolated from surface-sterilized roots (9 isolates) and leaves (12 isolates) of *E. vesicaria* ssp*. sativa* plants. Out of the 36 bacterial isolates, 9 endophytic isolates (2 isolates from roots and 7 isolates from leaves) could produce myrosinase activity in the range of 0.08 ± 0.03 to 19.92 ± 1.23 U mL^−1^ (Table 1). However, all rhizospheric isolates failed to produce any myrosinase activity.

**Table 1:** List of myrosinase-producing bacteria used in this study and their taxonomical identification

Isolate	Source	Myrosinase activity* (U mL^−1^)	16S rRNA		Identity based on 16S rRNA gene sequence	
			Length (bp)	NCBI access	Closest species to EZbiocloud database	Identity (%)
NGB-B9	Leaf	5.92 ± 0.31	1337	LC589981	*Bacillus siamensis* KCTC 13613^T^ (AJVF01000043)	99.93
NGB-B10	Leaf	19.92 ± 1.23	1308	LC589982	*Bacillus siamensis* KCTC 13613^T^ (AJVF01000043)	99.46
NGB-B11	Leaf	6.71 ± 2.08	1086	LC589983	*Bacillus velezensis* CR-502^T^ (AY603658)	99.91
NGB-B12	Leaf	4.43 ± 0.51	1346	LC589984	*Bacillus siamensis* KCTC 13613^T^ (AJVF01000043)	99.93
NGB-B14	Leaf	10.14 ± 0.17	1358	LC589985	*Bacillus velezensis* CR-502^T^ (AY603658)	99.63
NGB-B15	Leaf	0.08 ± 0.03	1301	LC589986	*Bacillus siamensis* KCTC 13613^T^ (AJVF01000043)	99.62
NGB-B17	Leaf	5.75 ± 1.79	1418	LC589987	*Bacillus siamensis* KCTC 13613^T^ (AJVF01000043)	99.58
NGB-B21	Root	1.32 ± 0.06	1357	LC589988	*Bacillus siamensis* KCTC 13613^T^ (AJVF01000043)	99.04
NGB-B26	Root	9.65 ± 1.03	1362	LC589989	*Bacillus siamensis* KCTC 13613^T^ (AJVF01000043)	99.93

*Data are presented as the mean of triplicate and are followed by the standard deviation (± SD)

### Bacterial identification

**Fig. 2:**
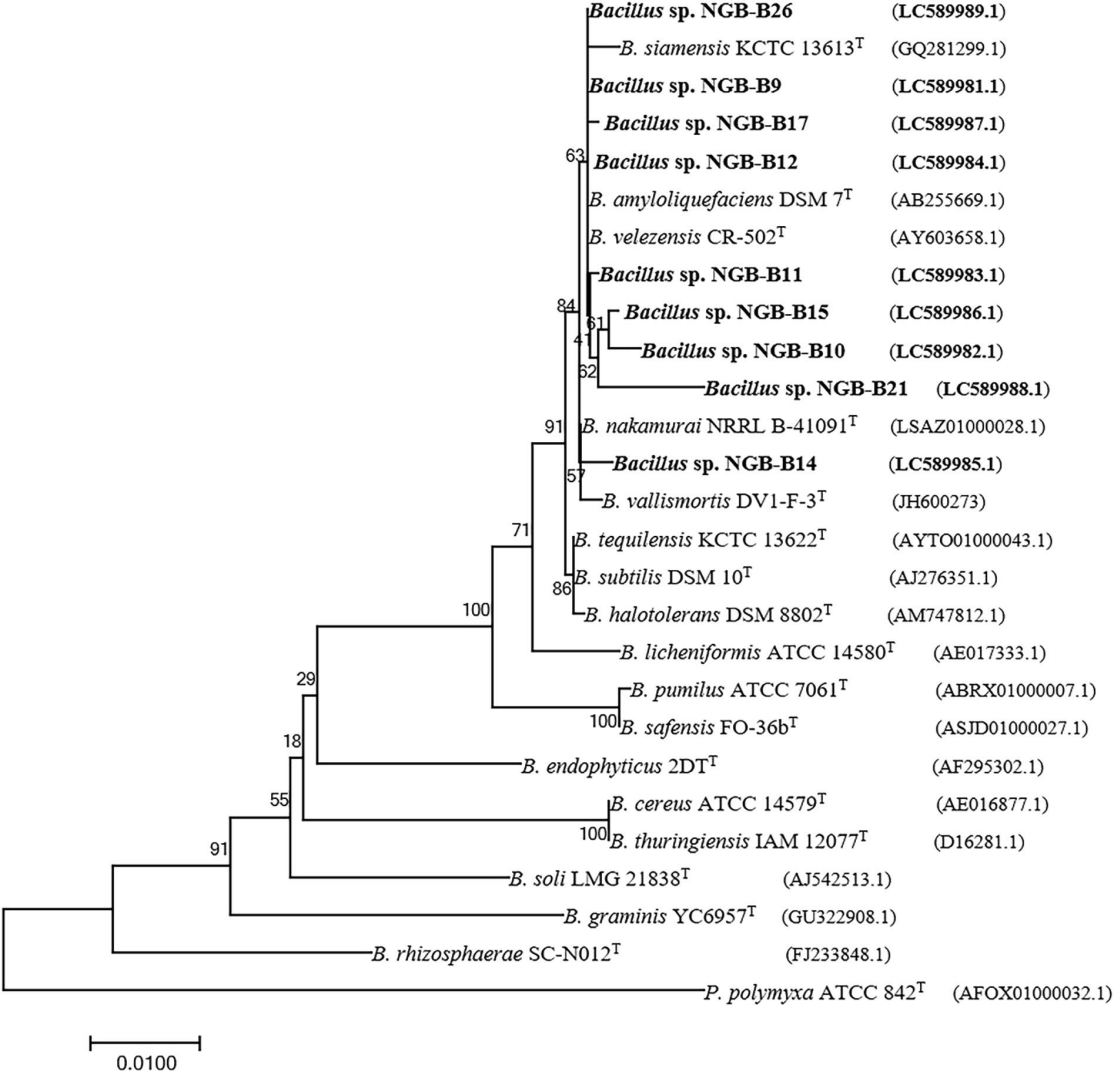
The phylogenetic relationships between myrosinase-producing bacterial isolates (bold) and reference strains based on 16S rRNA gene sequences. NCBI accession numbers are in parentheses. Bootstrap values are indicated for each node (1000 replicates). *B* (*Bacillus*), *P* (*Paenibacillus*), *NGB* (National Gene Bank)

The 16S rRNA genes from the 9 endophytic bacteria with myrosinase activity were sequenced and deposited (accessions: LC589981-LC589989) in the Genbank database (www.ncbi.nlm.nih.gov). The sequences of the 16S rRNA gene were blasted to the EZBioCloud database (https://www.ezbiocloud.net/) (Table 1). Isolates NGB-B11 and NGB-B14 shared 99.91 and 99.63% similarity, respectively, with the type strain *Bacillus velezensis* CR-502^ T^. While, the rest of the isolates had 99.04–99.93% 16S rRNA sequence similarity with the type strain *Bacillus siamensis* KCTC 13613^T^, a plant growth-promoting bacterium with antimicrobial compounds against plant pathogens (Jeong et al. 2012). According to the ML-phylogenetic tree based on 16S rRNA sequences (Fig. 2), all bacterial isolates were classified into the genus *Bacillus*. All myrosinase-producing bacteria were clustered with five species of the genus *Bacillus*: *B. amyloliquefaciens*, *B. nakamurai*, *B. siamensis*, *B. vallismortis*, and *B. velezensis*. Due to the low phylogenetic power at the species level, the newly isolated bacteria in this study were assigned only to the genus level and were identified as *Bacillus* sp.

### Identification of *bglA* myrosinase-related gene

The putative myrosinase gene from *Bacillus* sp. NGB-B10 was amplified (650 bp) using a PCR with designed primers, sequenced, and deposited in the GenBank database (accession: LC703353). The translated protein (accession: BDH43810) of the resulting myrosinase sequence revealed a 96.0% (e value 1e−119) sequence similarity to the reference sequence of 6-phospho-β-glucosidase (*bglA*) (accession: WP_064107630.1) of *B. amyloliquefaciens* group (*B. amyloliquefaciens*, *B. velezensis*, and *B. siamensis*). For microorganisms possessing myrosinase activity, the tblastn alignment revealed a 73.14% (e value 3e–90) sequence similarity with the *bglA* from *E. coli* O157:H7 strain Sakai (accession: BA000007.2) and *E. coli* O157:H7 strain TW14359 (accession: CP001368.1). While, a 73.71% (e value 2e−89) sequence identity was found with *E. ludwigii* strain EcWSU1 (accession: CP002886.1). Interestingly, we found a 34.10% (e value 1e−32) and 36.36% (e value 2e−38) sequence identify with plant myrosinase (accession: AAG54074.1) and cabbage aphid myrosinase (accession: AAL25999.1), respectively. However, we did not find any sequence similarity with myrosinase from *Citrobacter* sp. strain WYE1 (KT821094.1) or *Leclercia adecarboxylata* strain G1 (Tie et al. 2021).

### Classical optimization of media components to enhance myrosinase production

**Fig. 3:**
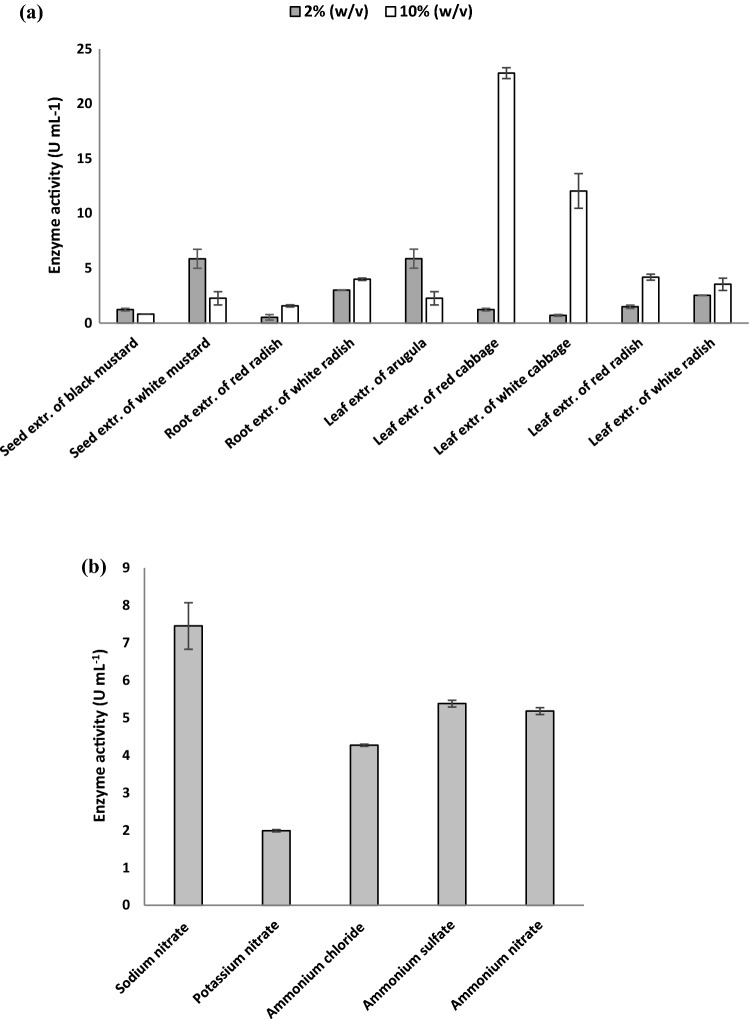
Effects of different carbon sources (**a**) and nitrogen sources (**b**) on myrosinase production from *Bacillus* sp. strain NGB-B10

To achieve optimal enzyme production by the highest myrosinase-producing bacterium (*Bacillus* sp. strain NGB-B10), various studies on medium composition had been carried out to select the optimum carbon and nitrogen sources by using the OFAT. The effects of different carbon (2 and 10% w/v) and nitrogen sources (200 mmol L^−1^) on the production of myrosinase by *Bacillus* sp. strain NGB-B10 are shown in Fig. 3a, b. Out of the 9 carbon sources investigated, the leaf extract of red cabbage exhibited the highest myrosinase activity (22.79 ± 0.50 U mL^−1^), followed by the leaf extract of white cabbage (12.04 ± 1.58 U mL^−1^) at a concentration of 10% (w/v). Generally, increasing the concentration of cruciferous plant extracts resulted in higher myrosinase yield except for leaf extract from arugula, and seed extract from black and white mustard. For nitrogen source, sodium nitrate was found to be the most effective for myrosinase production (7.45 ± 0.62 U mL^−1^) which was followed by ammonium sulfate (5.38 ± 0.09 U mL^−1^) and ammonium nitrate (5.18 ± 0.09 U mL^−1^). While potassium nitrate drastically decreased enzyme production (1.99 ± 0.03 U mL^−1^).

### Statistical medium optimization for myrosinase production

#### Plackett–Burman experimental design

A total of 7 parameters were screened for their effects on myrosinase production by *Bacillus* sp. strain NGB-B10 in 12 trials using the Plackett–Burman experimental design (Table 2). The maximum myrosinase production (21.93 ± 4.40 U mL^−1^) was attained in trial No. 2, while no myrosinase activity was detected in trials No. 3, 7, and 8 (Table 2). The Statistical analysis using the PBD (Tables 3, S3) indicated that nitrogen concentration (sodium nitrate), incubation period, and agitation speed significantly affected myrosinase production, while the other components were found to be insignificant. According to the design (Table S3), nitrogen concentration, temperature, pH and agitation speed have positive effects while three variables including the inoculum volume, the incubation period, and carbon source concentrations (leaf extract of red cabbage) have negative effects on the myrosinase activity. The interactive effects between selected critical components for maximum myrosinase production were further analyzed using the BBD of RSM.

**Table 2:** PBD for screening of significant factors affecting the myrosinase production by *Bacillus* sp. strain NGB-B10

Trial	Coded value							Enzyme activity (U mL^−1^)^*^	
	X_1_	X_2_	X_3_	X_4_	X_5_	X_6_	X_7_		
1	+	−	−	+	−	+	+	1.48	± 0.18
2	+	−	+	+	+	−	−	21.93	± 4.40
3	−	−	+	−	−	+	−	0.00	± 0.00
4	−	+	−	−	+	−	+	2.38	± 0.91
5	−	−	−	+	−	−	+	0.46	± 0.35
6	−	+	−	+	+	+	−	0.57	± 0.16
7	−	−	+	−	+	+	+	0.00	± 0.00
8	−	+	+	+	−	−	−	0.00	± 0.00
9	+	+	−	−	−	+	−	2.13	± 0.66
10	+	+	+	−	−	−	+	14.43	± 8.45
11	+	−	−	−	+	−	−	0.79	± 0.08
12	+	+	+	+	+	+	+	4.90	± 4.01

*Data are presented as the mean of triplicate and are followed by the standard deviation (± SD)

**Table 3:** Analysis of variance **(**ANOVA) for selected factors in PBD

Source	Sum of squares	*df*	*F* value	*p* value > *F*
Model	463.22	5	9.06	0.0484
A-Agitation	52.62	1	7.55	0.0709
B-N-source concentration	159.33	1	22.85	0.0174
C-Incubation time	55.14	1	7.91	0.0672
AB	110.51	1	15.85	0.0284
BC	85.63	1	12.28	0.0394
Error	20.92	6		
Corrected total	484.14	11		

R^2^ = 0.960241; adjusted R^2^ = 0.854219

#### Box–Behnken experimental design

Based on the PBD analysis, the BBD of RSM was applied to determine the optimal levels of the three selected variables (sodium nitrate, incubation period, and agitation speed). A total of 15 experiments with different combinations of the selected parameters at 3 levels were performed. The design matrix and the corresponding responses are presented in Table 4. The experimental results were analyzed by standard ANOVA. The BBD was fitted with the second-order polynomial equation:

$${\text{Y}} = { 89}.{348456614 } - { 2}.{54}0{\text{33488X}}_{{1}} + \, 0.{\text{3995476229X}}_{{2}} - { 8}.{\text{495391244X}}_{{3}} + \, 0.0{\text{216673352X}}_{{1}}^{{2}} + \, 0.000{\text{8962435X}}_{{2}}^{{2}} - \, 0.00{93}0{\text{1295 X}}_{{1}} {\text{X}}_{{2}} + \, 0.{\text{1567843728X}}_{{1}} {\text{X}}_{{3}}$$

where Y is the predicted myrosinase production, X_1_, X_2_ and X_3_ correspond to incubation period, agitation speed, and sodium nitrate, respectively.

**Table 4:** Matrix and experimental results of BBD

Trial	N-source conc. (%)	Agitation (rpm)	Time (hours)	Enzyme activity (U mL^−1^)		
				Experimental		Predicted
1	0	0	0	28.86	± 1.71	28.52
2	0	0	0	28.83	± 2.02	28.52
3	0	− 1	1	20.93	± 0.98	24.35
4	− 1	0	1	2.49	± 0.51	− 1.03
5	− 1	− 1	0	17.79	± 0.79	17.14
6	1	1	0	25.23	± 0.33	57.74
7	1	0	1	9.31	± 0.71	9.58
8	− 1	0	− 1	63.06	± 0.20	86.53
9	0	1	− 1	135.68	± 1.44	131.49
10	0	− 1	− 1	30.65	± 0.40	31.40
11	− 1	1	0	57.06	± 1.04	61.43
12	1	0	− 1	65.11	± 0.14	68.54
13	0	1	1	13.01	± 0.56	12.83
14	1	− 1	0	16.98	± 0.18	13.45
15	0	0	0	28.06	± 0.84	28.52

**Fig. 4:**
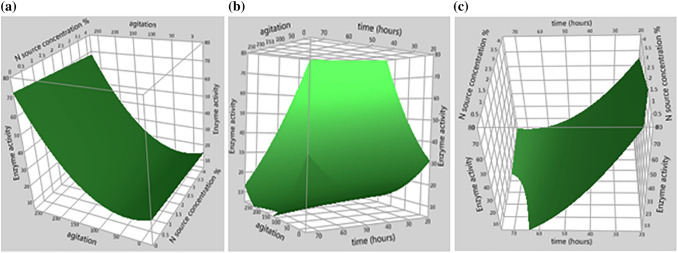
3D response surface plot for myrosinase production from *Bacillus* sp. strain NGB-B10 with the interaction between **a** N-source (sodium nitrate) concentration with agitation, **b** agitation with cultivation time and **c** cultivation time with N-source concentration

The statistical analysis of obtained data revealed that the model *F* value was 120.68 with a low probability value (*p* < 0.0001), indicating that the proposed model is highly significant (Table 5). The *R*^*2*^ value of the model was 0.976368 and the adjusted *R*^2^ value of the model was 0.994116 demonstrating the fitting of the tested model. Three-dimensional response surface curves were plotted to explain the interaction effect of the variables and to determine the optimum level of each variable for maximum response (Fig. 4a–c). Each figure demonstrated the effect of two factors while the third factor was fixed at the zero level (intermediate level). The optimum conditions for myrosinase production by *Bacillus* sp. strain NGB-B10 were determined using the response surface analysis and regression equation. The predicted values of the regression equation were generally consistent with the experimental values obtained by three repeated experiments. At the optimum experimental conditions, the maximum myrosinase activity was found to be 135.68 ± 1.44 which was very close to the response predicted by the regression model verifying the validity of the model.

**Table 5:** Analysis of variance **(**ANOVA) of BBD response variables

Source	Sum of squares	*df*	*F* value	*p* value > *F*
Model	14,604.739	7	120.6845	< 0.0001
A-Agitation speed	886.03	1	51.25	0.0008
B-N-source concentration %	94.55	1	5.47	0.0665
C-Cultivation time	399.91	1	23.13	0.0048
A^2^	415.25	1	24.02	0.0045
C^2^	391.52	1	22.65	0.0051
AC	3144.47	1	181.89	0.0001
BC	107.91	1	6.24	0.0546
Error	86.440	5		
Lack of fit	86.02991	3	139.9017	
Pure error	0.409954	2		
Corrected total	14,691.178	12		

Model R^2^ = 0.994116; Model adjusted R^2^ = 0.985879, Model mean of response = 34.9815

Based on the surface response graphs and the regression analysis, we found that the optimal conditions to achieve the maximum production of myrosinase enzyme were at 2.25 g L^−1^ sodium nitrate, 9.4 h of the incubation period, and 250 rpm. At these optimized conditions, the predicted enzyme activity was found to be 199.848 U mL^−1^ with a 10.03-fold enhancement as compared to the initial production medium (19.92 U mL^−1^).

### Purification of myrosinase

**Fig. 5:**
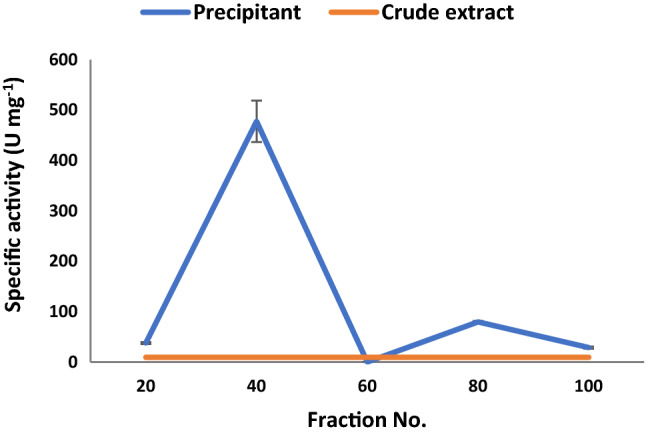
Specific activity of myrosinase from *Bacillus* sp. strain NGB-B10 that was recovered at different ammonium sulfate fractions compared to the crude extract

**Fig. 6:**
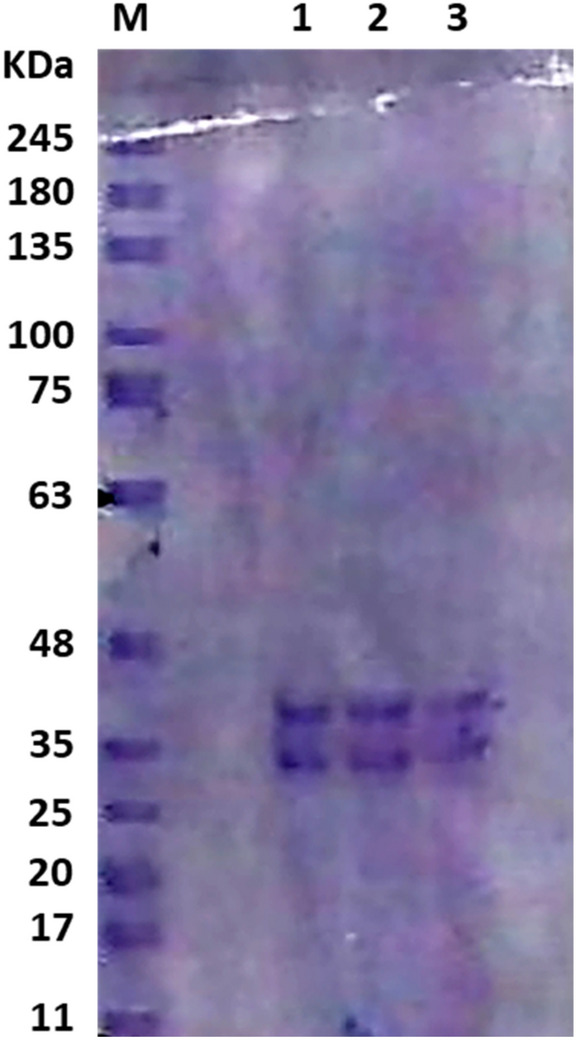
SDS–polyacrylamide gel electrophoresis of myrosinase purified by ammonium sulfate precipitation. M: Standard protein marker (GangNam˗Stain™ pre˗stained protein ladder). Lanes 1–3: precipitated myrosinase at a 40% fraction

Myrosinase was purified from the culture media of *Bacillus* sp. strain NGB − B10 by ammonium sulfate fractionation (20–100%). The results showed that the 40% fraction was the most adequate amount of salt to precipitate the myrosinase, as 477.6 U mg^−1^ protein of the enzyme activity was recovered in the precipitate (Fig. 5). However, the rest of the fractions had a lower efficiency for myrosinase precipitation (0.0–79.38 U mg^−1^ protein). The specific activity of crude enzyme extract increased from 2.1 to 477.7 (227.5-fold) with an 89.8% recovery of myrosinase activity by the ammonium sulfate precipitation (Table 6). Considering the higher enzymatic recovery of the 40% fraction, the purified enzyme in this fraction was analyzed using SDS-PAGE, and two distinct bands were yielded on the gel (Fig. 6). In addition, Log M_W_ vs. Relative Mobility (R_f_) graph was plotted which showed that the enzyme subunits had an approximate molecular weight of 38.6 and 35.0 KDa (Fig. S1).

**Table 6:** Summary of protein and myrosinase purification from *Bacillus* sp. NGB-B10 using ammonium sulfate precipitation

	Volume (mL)	Total protein (mg)	Total activity (U mL^−1^)	Specific activity (U mg^−1^)	Purification fold	Yield (%)
Crude extract	940	83,481	179,164	2.1	1	100
Ammonium sulphate	15	337	160,847	477.7	227.5	89.8

### Characterization of partially purified myrosinase

**Fig. 7:**
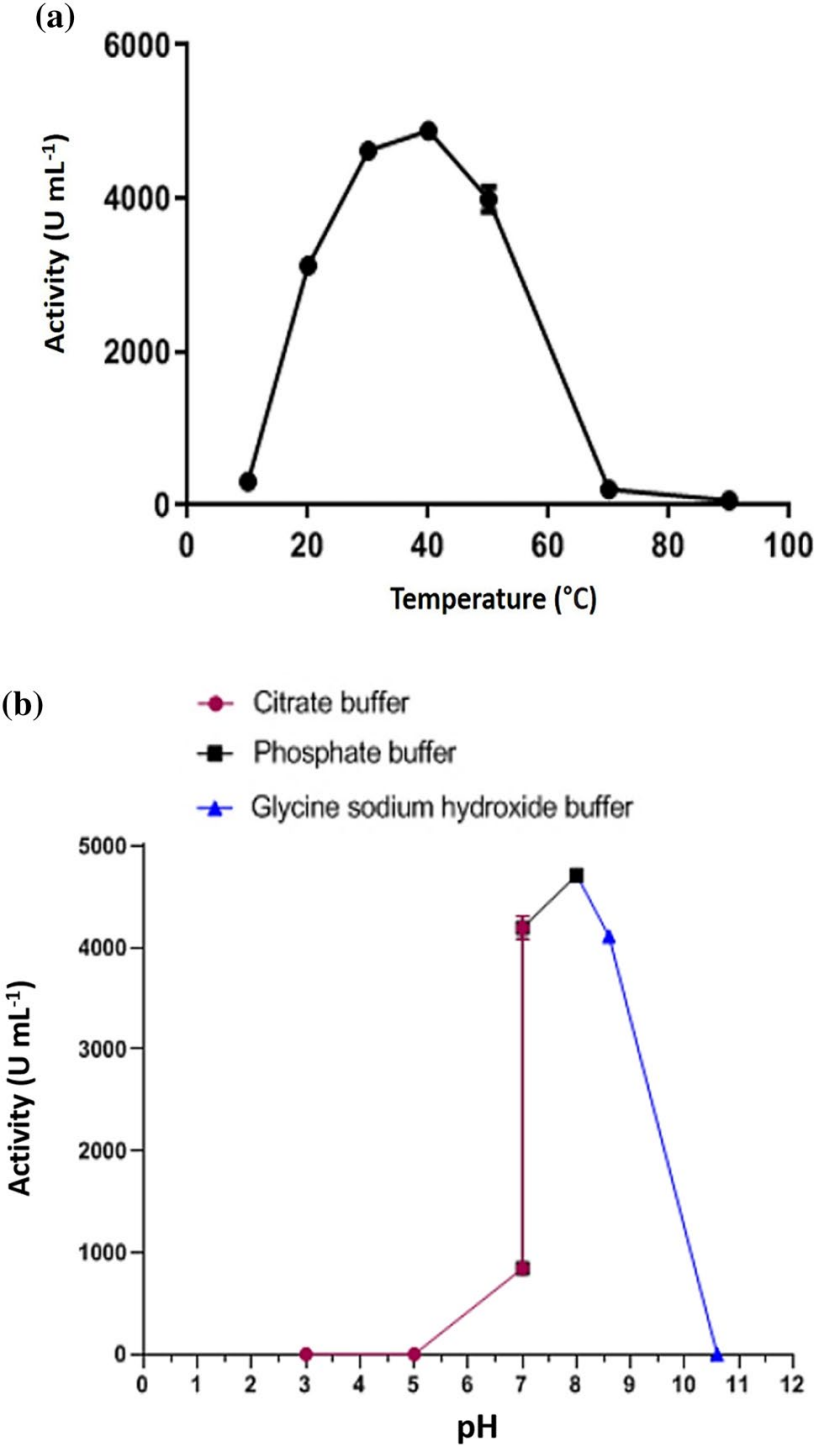
Effects of **a** temperature and **b** pH on the activity of the purified myrosinase from *Bacillus* sp. strain NGB-B10. Data are average values of three replicates, ± standard deviation (SD)

The influence of temperature on the activity of purified myrosinase is shown in Fig. 7a. The results indicated that there was no activity below 10 °C and it increased as the incubation temperature increased until it reached the highest enzyme activity at 40 °C. The activity then declined rapidly at temperatures higher than 40 °C until completely disappear at 70 °C. The effect of pH on the myrosinase activity was evaluated using different buffer solutions (Fig. 7b). The enzymatic activity gradually increased as the pH increased from pH 5.0 until it reached its maximum at pH 8.0. The activity then steadily decreased until it was completely inactivated at pH 10.5. The K_m_ and V_max_ parameters were determined to understand the reaction kinetics between purified myrosinase and different concentrations of the substrate (sinigrin). According to the resulting Lineweaver–Burk plot, the purified myrosinase demonstrated a K_m_ value of 2.5 mM and a V_max_ value of 5 mM sinigrin min^−1^ mg^−1^ (Fig. S2).

### Antifungal activity of GLs-myrosinase system

**Fig. 8:**
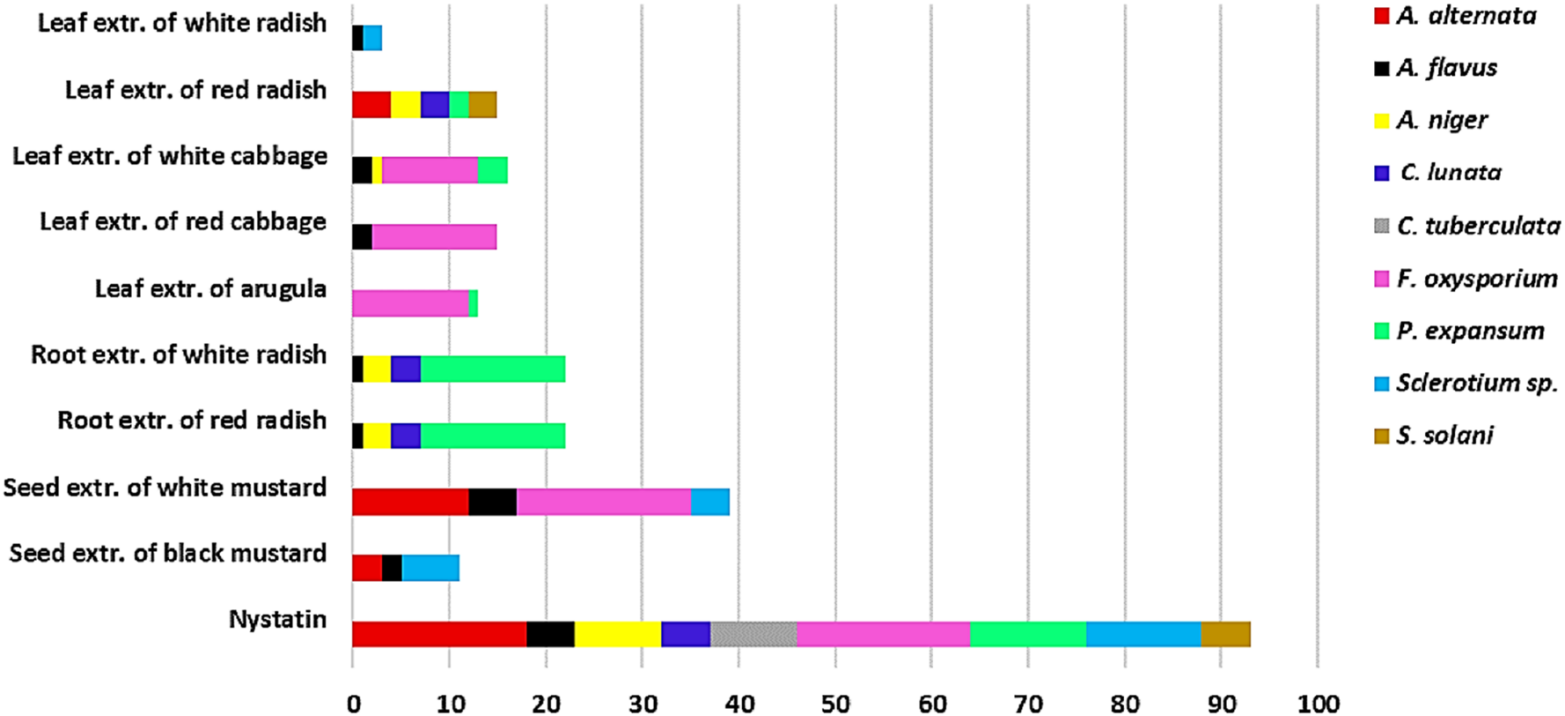
A stacked column chart expressing the antifungal activities of hydrolysis products released from different natural GLs extracts against phytopathogenic fungi. Data are expressed in mm of inhibition zone and are average values of three replicates

The growth inhibition of different phytopathogenic fungi growing on Czapek Dox agar medium and exposed to GLs-hydrolysis products was estimated using the agar well diffusion method, and the results had been expressed in mm as diameters of inhibition zones (Fig. 8 and Table S4). The results showed that GLs-hydrolysis products from various plant sources varied in their antifungal activities against tested fungal pathogens except for *C. tuberculate* which was resistant to all GLs extracts. It is worth mentioning that, hydrolytic products in GLs extracts from some *Brassicaceae* species had higher or equivalent inhibitory effects to nystatin (a broad-spectrum antifungal agent). For example, the antagonistic effects of GLs-hydrolysis products in seed extract of white mustard against *A. flavus* and *F. oxysporium* were similar to those obtained by nystatin. Also, the antifungal activities of GLs-hydrolysis products released from the root extract of both red radish and white radish towards *P*. *expansum* were greater than those recorded by nystatin. Out of all tested phytopathogenic fungi, *A. flavus* was the most sensitive which was inhibited by most (7/9) of investigated extracts.

## Discussion

A hallmark trait of the *Brassicaceae* family is their ability to produce GLs that can be hydrolyzed into glucose and bioactive products via the myrosinase enzyme (Kissen et al. 2009). The GLs-myrosinase system has been well studied in plants (Bhat and Vyas 2019) but little research has been done on the metabolism of GLs by microbial myrosinase (Narbad and Rossiter 2018). Arugula (*E. vesicaria* ssp*. sativa*) like other cruciferous vegetables produces diverse secondary metabolites, particularly the GLs which can influence its associated microbiota. This study used a cultivation-dependent approach to isolate GLs-degrading bacteria associated with arugula plants. All GLs degraders were only endophytic bacteria and were identified as *Bacillus* sp. using the 16S rRNA sequence analysis. The genus *Bacillus* has been previously described as an endophyte of many *Brassica* vegetables (Card et al. 2015). However, few studies have reported the isolation of strains belonging to Bacilli that are capable of in-vitro metabolism of GLs, for example, the ability of four *Bacillus* isolates to hydrolyze both sinigrin and other natural glucosinolates from rapemeal has been previously documented (Brabban and Edwards 1994). Diverse species of the genus *Lactobacillus* were also identified as GLs-degrading microorganisms (Palop et al. 1995; Sikorska-Zimny and Beneduce 2021).

Although there are a large number of reports on bacteria with myrosinase-like activities that can metabolize GLs and produce bioactive compounds (Narbad and Rossiter 2018), identification of the myrosinase enzyme in these microorganisms is still limited to only a few studies. It is now documented that, myrosinase metabolic activity is not restricted to a single phylotype or a family of bacterial species (Albaser et al. 2016; Wassermann et al. 2017; Tie et al. 2021; Cebeci et al. 2022). In the present study*,* we isolated the *bglA* gene, one of the genes responsible for myrosinase activity, from *Bacillus* sp. strain NGB-B10. Protein analysis using InterPro (https://www.ebi.ac.uk/interpro/) revealed that the *bglA* identified in this study belongs to the GH1 family which is similar to some myrosinases of bacterial origin (Cordeiro et al. 2015; Wassermann et al. 2017). The *bglA* gene was previously detected in *E. cloacae* strain KS50, an active myrosinase-producing bacterium isolated from the endosphere of turnip cabbage (Wassermann et al. 2017)*.* Furthermore, the ability *E. coli* strain 0157:H7, a high active GLs-metabolizing bacterium, to metabolize sinigrin was significantly reduced when the *bglA* was disrupted (Cordeiro et al. 2015)*.* However, the *bglA* of the current study differs from other bacterial myrosinases belonging to glycoside hydrolase family 3 (GH3) which have been identified from *Citrobacter* sp. strain Wye1 (Albaser et al. 2016; Cebeci et al. 2022) and *L. adecarboxylata* strain G1 (Tie et al. 2021). It has been reported that myrosinase enzymes corresponding to GH1 and GH3 differ in their properties and mechanisms of hydrolysis (Tie et al. 2021). Taken together, our data indicated that the GLs-metabolizing mechanism of *Bacillus* sp. NGB-B10 appears to be similar to that of *E. coli* strain 0157:H7, not *Citrobacter* strain WYE1 or *L. adecarboxylata* strain G1. It is noteworthy that the detected similarity between *bglA* from *Bacillus* sp. NGB-B10 and both plant myrosinase and cabbage aphid myrosinase could be largely due to both of them falling into the glycoside hydrolase family 1 (GH1) (Husebye et al. 2005; Lv et al. 2022)*.*

Previous studies showed that K_m_ values, using sinigrin as a substrate, varied according to the type of myrosinase (Hang 1994; Jones et al. 2001; Pontoppidan et al. 2001; Andersson et al. 2009). Compared to other myrosinases of microbial origin, the K_m_ value of the myrosinase enzyme identified from *Bacillus* NGB-B10 has a lower affinity for sinigrin than the cymr myrosinase identified from *Citrobacter* sp. strain WYE1 (Albaser et al. 2016; Cebeci et al. 2022). Similarly, it has a lower affinity for sinigrin than the plant myrosinase (Li and Kushad 2005) and aphid myrosinase (Husebye et al. 2005). While it has a higher affinity for sinigrin compared to fungal myrosinase identified from *A. niger* AKU 3302 (Ohtsuru and Hata 1973). We do not have a clear explanation for this inconsistency that is reflected in the literature, but it could be largely attributed to the source of the myrosinase, the type of substrate, or the experimental conditions.

The production of enzymes by microorganisms depends on the availability of carbon and nitrogen sources (Mefteh et al. 2019). In the current study, various sources of carbon and nitrogen were investigated to reach the maximum myrosinase production by *Bacillus* sp. strain NGB-B10. We found that the leaf extract of red cabbage and sodium nitrate were the best sources of carbon and nitrogen, respectively. These results are consistent with the previous studies indicating the use of natural extracts of *Brassica* plants as carbon sources to induce myrosinase production (Rakariyatham et al. 2005; Szucs et al. 2018). The high efficiency of leaf extract of red cabbage to increase myrosinase production may be attributed to its high content of several GLs (Wu et al. 2021). Stimulation of the activity of various bacterial enzymes by sodium nitrate as a nitrogen source has been also reported in previous studies (Rajoka 2004; Sereen et al. 2015).

The use of statistical models such as RSM to optimize the production of microbial enzymes and important metabolites has been successfully applied in recent research, due to the applicability and suitability of this approach for a wide range of studies (Mefteh et al. 2019; Ahmad et al. 2020). To the best of our knowledge, this is the first report which gives a detailed study of the application of RSM for myrosinase production from bacteria. Based on the Plackett–Burman experimental design, sodium nitrate concentration, incubation period, and agitation speed were the significant variable affecting myrosinase production from *Bacillus* sp. strain NGB-B10. The Box–Behnken experimental design of RSM was then applied to address the optimum levels of each significant parameter and their interactive effects on myrosinase yield. The application of RSM resulted in a myrosinase activity with a 10.03 increase higher than that obtained before optimization. Various reports documented that nitrogen source has a major effect on regulating bacterial growth and enzyme synthesis (Mefteh et al. 2019; Sahu et al. 2020). The incubation period is also a key parameter influencing the production of bacterial enzymes in culture media or submerged fermentation which is correlated to the reduction of nutrients available to the bacterial cells (Sahu et al. 2020). Furthermore, agitation speed plays an important role in promoting cell growth and thus enzymatic production which is most likely due to the high oxygen supply and nutrient transfer rate in the culture medium (Sai-Ut et al. 2014).

The environmental conditions especially the temperature and pH have a significant role in the induction or inactivation of enzyme activity. Experimental results indicated that the myrosinase enzyme from *Bacillus* sp. strain NGB-B10 could work at a broad range of pH (5.5–10) and temperatures (10–65 °C), which increases the possibility of its application under various conditions. In contrast with other bacterial myrosinases whose maximal activities occur at 25 °C (Tie et al. 2021; Cebeci et al. 2022), the optimum temperature for myrosinase activity in this study was found to be 40 °C which is consistent with the activity observed for some myrosinases of plant origin (Andersson et al. 2009; Bhat et al. 2015). We do not have a clear explanation for the high thermal stability of myrosinase under this investigation which is higher than that reported so far for other microbial myrosinases, even those purified from fungi (Rakariyatham et al. 2005). However, it can be attributed to the nature of its source, as the genus *Bacillus* is known as a potential source of producing thermostable enzymes (Cotta et al. 2021; Aktayeva et al. 2022). Additionally, the myrosinase in this study appears to prefer alkaline conditions (with an optimal pH of 8.00) compared to myrosinases previously isolated from other bacteria that had optimal pH values in the range of 6.0–6.6 (Tie et al. 2021; Cebeci et al. 2022). Similar to our findings, higher optimal pH values ranging from 6.0 to 7.5 were reported for myrosinases purified from different fungal strains corresponding to *Aspergillus* sp. (Rakariyatham et al. 2005). Although there was no clear evidence, we suggested that it could be related to the alkaline nature of the Egyptian soils where the host plant of *Bacillus* sp. NGB-B10 was cultivated. In agreement with our hypothesis, (Cebeci et al. 2022) suggested that the pH stability profile of myrosinase isolated from *Citrobacter* sp. strain Wye1 might be correlated to the soil pH value where that bacterium was isolated.

The GLs-hydrolysis products have important impacts on plant protection (Wassermann et al. 2017; Arroyo et al. 2019). In this context, the experimental results demonstrated that GLs-hydrolysis products from various cruciferous plants released by the myrosinase from *Bacillus* sp. NGB-B10 had diverse antagonistic effects against several phytopathogenic fungi. These various antifungal activities may be related to the presence of different GLs contents in cruciferous plants (Bhandari et al. 2015; Rhee et al. 2020), which leads to the release of diverse hydrolytic bioactive compounds (Plaszkó et al. 2021). The high activities observed in the case of white and red radish root extracts against *P*. *expansum* could be largely due to their high GLs content of glucoraphasatin and glucoraphanin (Luo et al. 2022), the precursors of ITCs such as raphasatin and sulforaphane which have strong antimicrobial activities (Kim et al. 2015; Abukhabta et al. 2021). Whereas, the high antagonistic effects of white mustard seed extract against *A. flavus* and *F. oxysporium* are likely attributed to the hydrolysis products of sinalbin, the most common GLs in white mustard seeds and the precursor of benzyl-ITCs (Lietzow 2021) which are known as potent antifungal agents (Wang et al. 2020). Notably, in the current study, the GLs-hydrolysis products from all investigated cruciferous plants had no inhibitory effects against *C. tuberculate* fungus. This could be explained by the ability of some pathogenic microorganisms such as *Sclerotinia sclerotiorum* to detoxify GLs-hydrolysis products via enzymatic hydrolysis and therefore exhibit resistance to the GLs-myrosinase system (Chen et al. 2020).

## Conclusion

The present study showed that bacterial endophytes of *Brassica* vegetables such as arugula are key participants in the GLs-myrosinase system and afford a new source of active microbial myrosinase. We identified the gene encoding this GH1 myrosinase and studied some characteristics of the purified enzyme. RSM was an effective tool for improving myrosinase production and resulted in a 10.03-fold enhancement in enzyme activity. The present work provides preliminary data that confirmed the presence of bioactive compounds in the GLs-hydrolysis products that have potent antifungal activities against several phytopathogenic fungi. These findings offer new sustainable approaches for plant protection by using *Brassica* endophytes. However, further studies are required to scale-up myrosinase production to control plant pathogens. Future perspectives of this study also include structural analysis of this GH1-myrosinase as well as the chemical analysis of GLs-hydrolysis products and study of their mode of action against plant pathogens.

## Supplementary Information

Below is the link to the electronic supplementary material.

Supplementary file1 (DOCX 414 kb)
