# Supplementary material for: A new source of bacterial myrosinase isolated from endophytic Bacillus sp. NGB-B10, and its relevance in biological control activity
Source: World J Microbiol Biotechnol. 2022 Sep 3;38(11):215. doi: 10.1007/s11274-022-03385-3 (PMC9440883; doi:10.1007/s11274-022-03385-3)
Supplement: Supplementary file 1 — Supplementary file1 (DOCX 414 kb) [file 11274_2022_3385_MOESM1_ESM.docx]

**Supplementary file**

**Table S1** Variables and levels for Plackett Burman experiment

| Variable | Variable code | Low level (-) | High level (+) |
| --- | --- | --- | --- |
| Agitation | X_1_ | Static conditions | 150 rpm |
| C-source (leaf extract of red cabbage)  concentration (%) | X_2_ | 4% | 20% |
| N-source (sodium nitrate)  concentration (%) | X_3_ | 0.2%  (23.5 mmol L^-1^) | 2%  (235.3 mmol L^-1^) |
| Temperature (ºC) | X_4_ | 25 ºC | 40 ºC |
| pH | X_5_ | 6.0 | 8.8 |
| Incubation period (days) | X_6_ | 1 | 5 |
| Inoculum size (mL) | X_7_ | 0.5 (10^7^ mL^-1^) | 5.0 (10^7^ mL^-1^) |

**Table S2** Significant factors for Box-Behnken experimental design and their tested levels

| high  (+1) | Intermediate  (0) | Low  (-1) | Factors |
| --- | --- | --- | --- |
| 72 | 48 | 24 | Incubation period (hours) |
| 250 | 150 | 0 | Agitation (rpm) |
| 4%  (470.6 mmol L^-1^) | 2%  (235.3 mmol L^-1^) | 0.2%  (23.5 mmol L^-1^) | N-source concentration  Sodium nitrate (%) |

**Table S3** Statistical significance of tested variables for production of myrosinase by *Bacillus* sp. strain NGB- B10 using Plackett-Burman experimental design

| Source | length of *t*-ratio | contrast | *p*-value | significance |
| --- | --- | --- | --- | --- |
| X_1_-Agitation | 2.33 | 3.52109 | 0.0410 | significant* |
| X_2_-C-source concentration (%) | -0.01 | -0.02103 | 0.9886 | non-significant |
| X_3_-N-source concentration (mmol L^-1^) | 1.85 | 2.78619 | 0.0814 | significant** |
| X_4_-Temperature (ºC) | 0.53 | 0.80033 | 0.6399 | non-significant |
| X_5_-pH | 0.67 | 1.00556 | 0.5315 | non-significant |
| X_6_-Incubation period (days) | -1.71 | 2.57661 | 0.0990 | significant** |
| X_7_-Inoculum size (mL) | -0.10 | -0.14789 | 0.9324 | non-significant |
| X_1_X_3_ | 1.94 | 2.92708 | 0.0714 | significant** |
| X_1_X_6_ | -0.19 | -0.29326 | 0.8648 | non-significant |
| X_3_X_6_ | -1.72 | -2.58702 | 0.0981 | significant** |
| X_3_X_5_ | -0.1 | -0.15005 | 0.9317 | non-significant |

*,** Significant at *p* < 0.05 and *p* < 0.1, respectively

**Table S4** Antifungal activity of hydrolysis products released from different natural GLs extracts against phytopathogenic fungi

| Treatment | Tested fungal pathogen | | | | | | | | |
| --- | --- | --- | --- | --- | --- | --- | --- | --- | --- |
|  | 1. *alternata*   SCUF00001378 | *A. flavus*  SCUF00000113 | *A. niger*  SCUF0000087 | *C. lunata*  SCUF00000122 | *C. tuberculata*  SCUF0000055 | *F. oxysporium*  SCUF00000310 | *P. expansum*  SCUF00000332 | *Sclerotium* sp.  SCUF00000377 | *S. solani*  SCUF00000401 |
| **Nystatin (positive control)** | 18±0.5 | 5±1.0 | 9±1.0 | 5±1.0 | 9±1.0 | 18±0.7 | 12±1.0 | 12±0.5 | 5±1.0 |
| **Myrosinase**  **(negative control)** | - | - | - | - | - | - | - | - | - |
| **Seed extr. of black mustard** | 3±1.0 | 2±1.0 | - | - | - | - | - | 6±1.0 | - |
| **Seed extr. of white mustard** | 12±1.0 | 5±1.0 | - | - | - | 18±1.0 | - | 4±1.0 | - |
| **Root extr. of red radish** | - | 1±0.5 | 3±1.0 | 3±1.0 | - | - | 15±0.7 | - | - |
| **Root extr. of white radish** | - | 1±0.5 | 3±1.0 | 3±1.0 | - | - | 15±0.7 | - | - |
| **Leaf extr. of arugula** | - | - | - | - | - | 12±1.0 | 1±0.5 | - | - |
| **Leaf extr. of red cabbage** | - | 2±1.0 | - | - | - | 13±0.5 | 0 | - | - |
| **Leaf extr. of white cabbage** | - | 2±1.0 | 1±0.5 | - | - | 10±0.5 | 3±1.0 | - | - |
| **Leaf extr. of red radish** | 4±1.0 | - | 3±1.0 | 3±1.0 | - | - | 2±1.0 | - | 3±1.0 |
| **Leaf extr. of white radish** | - | 1±0.5 | - | - | - | - | - | 2±1.0 | - |

SCUF: Suez Canal University Fungarium. Data are expressed in mm of inhibition zone and are average values of three replicates. ± standard deviation (SD)


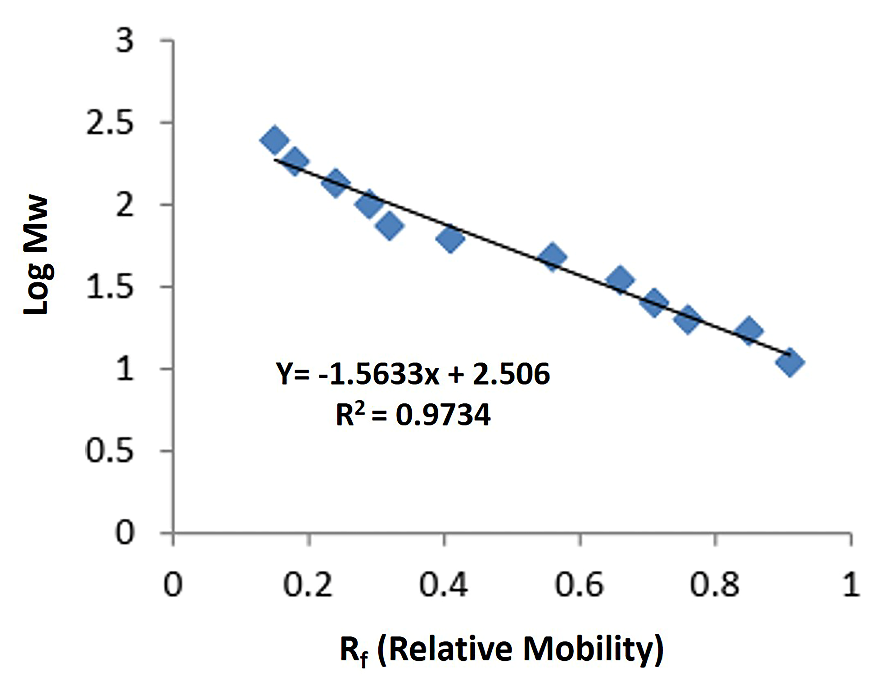


**Fig. S1** Standard Rf–log MW graph of myrosinase using the SDS-PAGE


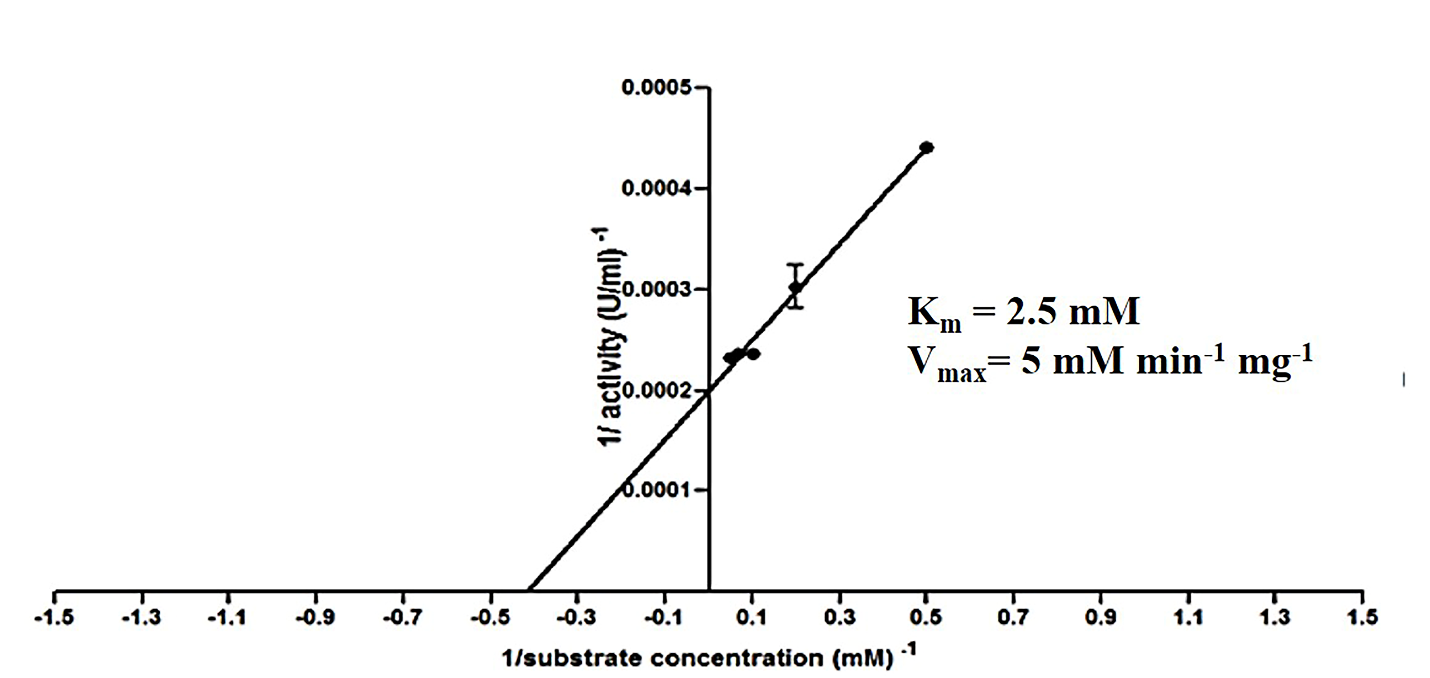


**Fig. S2** K_m_ and V_max_ values of purified myrosinase. (Lineweaver–Burke plot)
